# Essential tremor: the most common form of cerebellar degeneration?

**DOI:** 10.1186/s40673-020-00121-1

**Published:** 2020-08-14

**Authors:** Elan D. Louis, Phyllis L. Faust

**Affiliations:** 1 Department of Neurology and Therapeutics, University of Texas Southwestern, Dallas, TX USA; 2 Department of Pathology and Cell Biology, Columbia University Irving Medical Center and the New York Presbyterian Hospital, New York, NY USA

**Keywords:** Essential tremor, cerebellar degeneration, spinocerebellar ataxia, cerebellum, neurodegeneration, prevalence

## Abstract

**Background:**

The degenerative cerebellar ataxias comprise a large and heterogeneous group of neurological diseases whose hallmark clinical feature is ataxia, and which are accompanied, to variable degrees, by other features that are attributable to cerebellar dysfunction. Essential tremor (ET) is an exceptionally common neurological disease whose primary motor feature is action tremor, although patients often manifest intention tremor, mild gait ataxia and several other features of cerebellar dysfunction.

**Main Body:**

In this paper, we review the abundant evidence derived from clinical, neuroimaging and postmortem studies, linking ET to cerebellar dysfunction. Furthermore, we review the combination of clinical, natural history and postmortem features suggesting that ET is neurodegenerative. We then compare the prevalence of ET (400 – 900 cases per 100,000) to that of the other cerebellar degenerations (ranging from <0.5 – 9 cases per 100,000, and in composite likely to be on the order of 20 cases per 100,000) and conclude that ET is 20 to 45 times more prevalent than all other forms of cerebellar degeneration combined.

**Conclusion:**

Given the data we present, it is logical to conclude that ET is, by far, the most common form of cerebellar degeneration.

## Background

Degenerative cerebellar ataxias comprise a heterogeneous group of disorders whose hallmark clinical feature is ataxia (i.e., problems with force and timing of motion) and which are characterized on postmortem by various degrees of cerebellar degeneration; the list of such degenerative disorders of the cerebellum is extensive [1–7]. While the disorders are clinically heterogeneous, what they have in common is a related set of features that are referable to cerebellar dysfunction. Among several features, these can include gait ataxia, intention tremor, and eye movement abnormalities [1–6].

Although there are numerous forms of autosomal dominant and autosomal recessive spinocerebellar ataxias (SCAs), along with a plethora of other disorders of cerebellar degeneration that include both hereditary and acquired forms [2, 7], both individually and in composite, these disorders are characterized by modest prevalence [7–9].

The prevalence of the autosomal dominant SCAs has been estimated to be 1 - 9 cases per 100 000 people, and over 40 subtypes are now genetically defined [5, 8–15]. Among these, SCA2 and SCA3 are the most common subtypes worldwide; SCA1, SCA6, SCA7 and SCA8 often make up a relatively larger proportion of SCAs and the remainder tends to be rarer, although there is variation in prevalence from country to country [8, 10, 11, 13, 15]. Autosomal recessive ataxias are similarly characterized by a low prevalence, with Friedreich’s ataxia being the most common (prevalence = 0.5 – 5/100,000) [13, 16, 17]. Acquired degenerative ataxias are similarly rare – multiple system atrophy (3.4 – 4.3/100,000) [18, 19], paraneoplastic cerebellar degeneration (1.2/100,000) [20], and ataxia with vitamin E deficiency (0.06/100,000) [21].

Essential tremor (ET) is a common neurological disease [22]. Its primary motor feature is action tremor [23], although patients often manifest intention tremor [24–26], mild gait ataxia [27] and other features of subtle cerebellar dysfunction [28]. The pathophysiology is of ET unclear, and is likely heterogeneous. Recent work has recast it as one that is often due to cerebellar and cerebellar pathway dysfunction [28–36] as well as one that is likely degenerative [29, 37–51].

Although there are published scholarly reviews that have focused on the links between ET and the cerebellum [28, 30–32, 52, 53], other reviews that focused on its possible degenerative nature [29, 37, 39, 40, 47], and others that elucidated its high prevalence [22, 54], there is no work that has synthesized all of these loose threads and put them together in one place to support a new thesis – that ET is the most common form of cerebellar degeneration. The goals of this paper are to briefly review the evidence that ET is linked to the cerebellum, that ET is a degenerative disease, and that it has a very high prevalence. We then compare the prevalence of ET to that of other forms of cerebellar degeneration, as this has not yet been formally done. We then synthesize these different data streams to support the thesis that ET is the most common neurodegenerative disorder of the cerebellum.

## Methods

### Literature Review

One of the authors (E.D.L.) conducted a PubMed search on April 22, 2020. The goals of this search were to systematically identify papers that provided [1] data on the links between ET and the cerebellum, [2] data and discussion on the degenerative hypothesis of ET, and [3] data on the prevalence of ET. It was not a systematic search of all papers on disease mechanisms in ET, although the only other mechanistic disease model for ET is the olivary hypothesis, which has fallen out of favor in recent years [55–57]. During this search, the author searched abstracts of published papers, crossing the term “essential tremor” with a series of second terms, which yielded the following numbers of papers: essential tremor + cerebellum (*n =* 376), essential tremor + cerebellar (*n =* 459), essential tremor + degeneration (*n =* 124), essential tremor + degenerative (*n =* 63), essential tremor + neurodegeneration (*n =* 68), and essential tremor + neurodegenerative (*n =* 242). These papers were reviewed for relevant content and cited in this paper if relevant.

### Definitions

Prevalence is the proportion of the population having a disease, and it includes both crude and adjusted prevalence. Crude prevalence refers to the actual proportion of cases, and it may be contrasted with adjusted prevalence (e.g., age-adjusted prevalence), which is a proportion that has undergone statistical transformation to permit fair comparison with the prevalence in other groups that differ in some characteristic such as age or gender.

## Main Text

### High Prevalence of ET – Review of the Data

ET is one of the most common movement disorders and the most common form of tremor. A review of 28 population-based prevalence studies from 19 countries broadly examined the prevalence of ET globally [22]. In a meta-analysis, the prevalence among all ages was 0.9% (i.e., 900 per 100,000) [22]. In additional descriptive analyses, the crude prevalence among all ages was 0.4% (i.e., 400 per 100,000) [22]. Prevalence increased markedly with age, and especially with more advanced age [22]. In the meta-analysis, prevalence among persons age 65 years or older was 4.6% (i.e., 4,600 per 100,000), and in additional descriptive analyses, the median crude prevalence among those age 60 – 65 and older was 6.3% (i.e., 6,300 per 100,000) [22]. In one study of those age 95 and older, the crude prevalence was 21.7% (21,700 per 100,000) [22, 58]. In the United States alone, an estimated 7 million of individuals are affected, representing 2% of the entire population [54].

### ET – Review of its Links to the Cerebellum

In this section, we review the clinical, neuroimaging, and postmortem studies that point towards a link between ET and the cerebellum.

#### Clinical

A complete discussion of the clinical features that link ET to the cerebellum may be found elsewhere [28]. In approximately one-half of patients with ET, the kinetic tremor may have an intentional component [24]. This is of interest to the present discussion because intention tremor has canonically been viewed as a sign of cerebellar dysfunction. Thus, in these patients, during the finger-nose-finger maneuver, the tremor amplitude increases when the patient’s finger approaches his/her own nose or the examiner’s finger [24, 59, 60]. Furthermore, intention tremor is not restricted to the upper limbs in ET. Thus, an intention tremor of the head is seen in as many as 10% of ET patients (e.g., tremor occurs in the neck as the head moves towards a target, for example, while moving the neck forward to facilitate drinking from a cup) [61]. Intention tremor is also more prevalent in the legs of ET patients than in controls, observed in 1-in-4 ET patients [62].

Apart from tremor, a number of other motor features have been described in ET patients and these features point towards an underlying abnormality of the cerebellum or its projections. The most clinically-evident of these clinical features is gait ataxia, which is generally mild [27, 63–70]. In some ET patients, however, gait ataxia can be of moderate severity [71]. In a study of a 104 ET patients and 40 controls [72], in which patients were studied using quantitative gait analysis, ET gait was characterized under standard walking conditions by slower gait speed, problems with dynamic balance and gait temporal asymmetry. This constellation of impairments is similar to that which is seen in patients with cerebellar ataxic gait [27, 73–75]. Although the gait abnormality in ET is milder than that seen in patients with most forms of SCA, it does have clinical and functional consequences, with studies indicating that ET patients may exhibit reduced functional mobility both in terms of self-reported measures of gait confidence and in performance-based measures [27, 68, 69] and increased numbers of self-reported falls [69].

There are several other motor abnormalities that point to what is likely to be a more pervasive underlying abnormality of cerebellar function in ET. First, in a study of 14 ET patients and 11 controls, eye movements were recorded using a scleral search-coil technique, and vestibular function was assessed using electro-oculography [76]. ET patients demonstrated several oculomotor deficits that may have been indicative of cerebellar dysfunction - impaired initiation of smooth pursuit and pathological changes in the vestibulo-ocular reflex [76]. Of additional interest is that these oculomotor abnormalities were particularly evident among those ET patients who also exhibited intention tremor [76], which as noted above canonically has been viewed as a sign of cerebellar dysfunction. The presence of sub-clinical eye movement abnormalities in ET patients has been confirmed in several additional studies [77, 78]. Second, several studies have revealed abnormalities in limb motor behavior in ET [79–83]. Thus, a study of eye-hand coordination that compared 12 ET patients to 14 controls demonstrated abnormal kinematic changes during the early phase of pointing movements, which are also a feature of underlying cerebellar disease [81]. Another study compared 15 ET patients with 11 controls in terms of repetitive finger tapping movements [82]. ET patients demonstrated a longer touch duration, a lower inter-tapping interval and greater temporal variability when compared with controls [82]. During a predictive motor-timing task, which involved mediated interception of a moving target, some ET patients (i.e., the subgroup with head tremor) and patients with SCA were significantly worse at interception than were patients with Parkinson’s disease and controls, suggesting the presence of a fundamental problem with predictive motor timing [79, 83]. The regularity and the maximum frequency of auditory paced repetitive movements was also compared in 34 ET patients and 41 healthy controls - there was greater variability of rhythmic finger tapping and alternating hand movements in ET patients than controls, and timing of rhythmic movements of the two hands was disturbed to the same degree [80]. These results suggested the presence of a marked deficit of event-based rhythm generation in ET, and given the role of the cerebellum in motor timing [52, 84, 85], these provide further evidence of bilateral cerebellar dysfunction [80]. Third, motor learning also appears to be impaired in ET [86, 87]. In one study, investigators used conditioning of the eyeblink reflex to assess motor learning in 23 ET patients and 23 controls - the ability to acquire conditioned eyeblink responses was significantly reduced in ET [86]. In another study, investigators studied 10 ET patients and 9 controls using an eyeblink reflex conditioning paradigm, and motor learning was again abnormal in ET, again, likely reflecting pathological involvement of the cerebellum [87].

Aside from motor features, there is a growing literature that demonstrates the presence of non-motor features in patients with ET [88]. Among these is cognitive impairment, which may range from mild to marked [89]. While some of this impairment likely has its basis in degenerative pathology in the cerebral cortex [90, 91], some is also likely based in the cerebellum, as a cerebellar cognitive affective syndrome is well-known to occur and includes hallmark deficits in executive function [92]. That executive dysfunction is a common feature of the cognitive impairment of both ET and cerebellar-related cognitive dysfunction further suggests links between ET and the cerebellum [89].

In summary, as reviewed above, a range of clinical features link ET to a dysfunction of the cerebellar system. There are others. These include the observed resolution of ET in a patient after cerebellar stroke as well as the observation that cerebellar outflow pathways are the focus of several highly-effective surgical approaches to ET [28, 93]. As noted above, a detailed elaboration of the clinical features that link ET to the cerebellum may be found elsewhere [28].

#### Neuroimaging

Numerous neuroimaging studies have compared ET patients to controls [30, 34, 94–96]. Although there is some variation across studies, the most consistently involved brain structure is the cerebellum [30, 34, 94–96]. These studies have employed a broad array of methods, including magnetic resonance (MR) volumetry, MR spectroscopy, diffusion-weighted and diffusion tensor imaging, functional MR imaging, other MR imaging, and positron emission tomography (PET) [94]. This literature will be briefly reviewed.

In a comprehensive review published in 2014 [94], the data from the voxel based morphometry studies (i.e., MR volumetry) were somewhat mixed, yet the majority of studies (i.e., four of six [97–102]) reported a reduction in cerebellar volume in ET and, in particular, among the subgroup of ET patients who had head tremor. More recently, MRI volumetry analyses have reported similar reductions in specific cerebellar lobules in ET patients in comparison with controls [103].

MR spectroscopy data from two studies [104, 105] provide evidence that there is, at a minimum, neuronal dysfunction in the cerebellum in patients with ET, and quite possibly, neuronal loss [94].

In the 2014 review, seven studies [106–112] used diffusion imaging to compare ET patients to controls, using a variety of approaches, including a whole brain voxel-by-voxel comparison as well as a region of interest approach, targeting areas involved in the olivary–cerebellar–thalamic network [94]. Five of seven diffusion imaging studies demonstrated differences between ET cases and controls, and the two studies with null results had important methodological limitations [94]. More recent studies similarly show diffusion tensor changes in the ET vs. control cerebellum [51]. Hence, the bulk of evidence from the diffusion tensor imaging literature demonstrates that some orientation-dependent aspect of the microstructure of the tissue in ET is abnormal [94]. The bulk of these studies show evidence of axonal changes in the cerebellum, although more widespread changes have been noted as well [94].

In the comprehensive review [94], five studies were reported to have used functional MR imaging to study ET [113–117], with the first of these being a study of 12 ET patients and 15 controls in 1997, showing that ET is associated with an additional contralateral cerebellar pathway activation and overactivity in the cerebellum, red nucleus, and globus pallidus, without significant intrinsic inferior olivary nucleus activation [114]. Several of the other fMRI studies have similarly shown abnormalities in the cerebellar–thalamic network in patients with ET [113, 117]. More recent functional MRI studies have also detected changes in the ET cerebellar hemispheres and/or cerebellar vermis, among a limited number of other cortical and subcortical regions, in ET cases vs. controls [118, 119].

A sizable number of studies [120–125] have used PET to provide information about regional cerebral blood flow, regional glucose metabolism and regional binding of a variety of radiopharmaceutical compounds in patients with ET [94]. The most consistent finding in these studies is the localization of changes in blood flow to the cerebellum, although a number of other structures within the motor system, including the thalamus and red nucleus, have been implicated in selected studies as well. In another study [126], the authors performed ^11^C-flumazenil PET to calculate the distribution volume of the GABA_A_ complex, in eight ET cases and 11 controls. In ET, there was a significant increase in binding of ^11^C-flumazenil at the benzodiazepine receptor site of the GABA_A_ receptor in the cerebellum, the ventrolateral thalamus, and the lateral premotor cortex [126].

In summary, the most consistently involved structure in which abnormalities are seen in ET is the cerebellum, although a variety of studies also show abnormalities in other brain regions, many of which are part of the cerebellum’s tremor outflow pathway.

#### Pathological

Despite its extraordinarily high prevalence, the patho-mechanisms of ET have been elusive [47, 127–129]. Even the localization of the site of primary pathology within the brain has been a major challenge. In part, this was due to the lack of postmortem studies; indeed, during the 100-year period from 1903 to 2003, there were only 15 published postmortems [130], mainly comprising isolated case reports that lacked rigor as none used quantitative or immunohistochemical approaches nor compared ET to control brains [131–137]. A popular disease model, initially proposed in the 1970s [138], attempted to link ET to an abnormal inferior olivary nucleus; however, empiric support for that model is limited [57, 139, 140] and the model has recently fallen out of favor [55, 56]. More recent research in the field has focused on the cerebellum, where there has been a reconceptualization of the disease as one of cerebellar degeneration, as will be discussed below [29, 35, 37–47].

The neuropathology of ET has been reviewed in detail elsewhere [32, 47, 127, 128]; hence, selective comments will be made here. Studies over the past fifteen years have systematically identified a broad range of structural, degenerative changes in the ET cerebellum, spanning across all Purkinje cell compartments [47, 127, 128]. In the dendritic compartment, studies show an increase in number of Purkinje cell dendritic swellings [141], pruning of the dendritic arbor [142], and a reduction in spine density [142]. In the Purkinje cell somatic compartment, one sees a reduction in Purkinje cell linear density in some studies [143–145], an increase in empty basket cell processes (i.e., a marker of Purkinje cell loss) [146], and an increase in the number of heterotopically-positioned Purkinje cell soma [147, 148]. In the Purkinje cell axonal compartment, numerous changes in morphology have been observed, including an increase in the number of thickened axonal profiles, torpedoes, axonal recurrent collaterals, axonal branching, and terminal axonal sprouting [45, 149]. Additional changes, possibly due to secondary remodeling, have been observed in neighboring neuronal populations. These changes include a hypertrophy of basket cell axonal processes [150, 151] and changes in the distribution of climbing fiber-Purkinje cell synapses [45, 152, 153]. In carefully controlled studies, these changes all distinguish ET from normal control brains [45, 47]. Initial studies further indicate that the profile (i.e., constellation) of these changes may separate ET from other diseases of cerebellar degeneration, thereby serving as a preliminary disease signature, although these studies are in their infancy [32, 45, 47].

In the majority of ET cases, other than a subset of ET cases with Lewy bodies [144, 154], the postmortem changes that have been observed to date are exclusively in the cerebellum. Furthermore, the inferior olivary nucleus has appeared normal in postmortem studies, which echoes data from neuroimaging and clinical studies, which suggest that structural changes in this nucleus do not play a role in tremor generation in ET [55, 57, 125, 139, 140].

### ET as a Degenerative Disease – Review of the Data

There is growing support for the notion that ET is a neurodegenerative disease [29, 37–51]. Historically, the notion that ET could be neurodegenerative is not one that is new. As far back as 1948, Critchley and Greenfield wrote: “Although anatomical proof is as yet lacking, there are at least a number of clinical points to make question whether “essential tremor” may not, at times any rate, represent an incomplete or a premature variant of one of the cerebellar atrophies” [155]. Although not elaborated upon by those authors, these “clinical points” include the insidious onset of the disease, its association with advanced aging in the sense that both incidence and prevalence increase with aging exponentially, its gradual yet progressive nature, and the presence of numerous “cerebellar” features on neurological examination (e.g., intention tremor and ataxia, as discussed above). We will now elaborate on these and several additional points.

As is the case with other degenerative conditions such as Parkinson’s disease, SCA, or dementia, the onset of ET is similarly insidious, as has been well-documented [156, 157]. Indeed, age of onset can be difficult to determine because patients can have difficulty appreciating their tremor early on. Retrospectively recalling the point of onset can be similarly challenging, as tremor starts as one that is subtle, of low-amplitude, and difficult to distinguish from medication-induced tremor, anxiety-related tremor or other para-normal and/or situational forms of tremor [158–160]. As with other forms of neurodegeneration, the worsening of tremor in ET then follows a gradual yet progressive clinical course [161–163], albeit the pattern of progression differs across patients; there have never been any documented cases of reversion of tremor or cessation of disease once set in motion [39]. There is a marked and continued rise in disease occurrence (both incidence and prevalence), which is exponential in advanced ages [22, 58, 164–167], and which is yet another feature of the neurodegenerative conditions. Furthermore, ET itself has been associated with other neurodegenerative disorders. For example, there is a longstanding association between ET and PD [168–170]; indeed, having ET increases the risk of developing incident PD four to five-fold [171]. Furthermore, having older onset ET increases the risk of developing incident Alzheimer’s disease nearly two-fold [172, 173]. Similarly, a possible association between ET and another tauopathy, progressive supranuclear palsy, has been reported [174]. This association between ET and subsequent development of these neurodegenerative diseases suggests that ET could share pathogenic mechanisms with some of these disorders. Although not studied extensively, other features of neurodegenerative diseases (e.g., an olfactory deficit, increased risk of mortality) have also been associated with ET in a small yet relevant literature [40, 48, 175].

Neurodegenerative diseases have traditionally been characterized by the presence of selective involvement of anatomically and physiologically related systems of neurons, and neuronal loss is also considered by many to be a prominent feature of these diseases [40]. As seen in the degenerative ataxias, Purkinje cell loss has been noted in numerous studies of ET, with the extent of Purkinje cell loss being greater than seen in some forms of degenerative ataxia such as spinocerebellar ataxia 3 [45, 143–145]. In addition, as noted above, a host of other changes occur in the ET cerebellum, some of which are likely to be primary and degenerative, albeit many are on the milder end of the spectrum compared to other forms of SCA [45, 47].

As noted above, neurodegenerative diseases traditionally have been defined as diseases that begin insidiously and pursue a gradually progressive course that may continue for many years. Furthermore, their occurrence often increases markedly with advancing age. These and other clinical features, as well as postmortem features as reviewed above, suggest that ET is neurodegenerative. While many of these features in isolation are not specific to neurodegenerative diseases, the constellation of findings, all present in the same disease, is compelling. For a detailed review of these and other features, see Table 4 in Louis [40].

### Cumulative thesis: ET is the most common form of cerebellar degeneration

Given that [1] ET is highly prevalent, [2] clinical, neuroimaging and postmortem studies have linked ET to the cerebellum, and [3] clinical and postmortem features suggest that it is neurodegenerative, then one would conjecture that it might be the most common form of cerebellar degeneration. Before reaching this conclusion, we must compare the prevalence of ET with that of other cerebellar degenerations.

As noted above, the SCAs as a composite whole are characterized by modest prevalence [8, 9]. For example, the prevalence of the autosomal dominant SCAs has been estimated to be 1 - 9 cases per 100 000 people [8–15]. Autosomal recessive ataxias are similarly characterized by low prevalence; Friedreich’s ataxia, the most common, has a prevalence of 0.5 – 5/100,000 [13, 16, 17]. Acquired degenerative ataxias are similarly rare: multiple system atrophy (3.4 – 4.3/100,000) [18, 19], paraneoplastic cerebellar degeneration (1.2/100,000) [20], and ataxia with vitamin E deficiency (0.06/100,000) [21].

As noted above, the prevalence of ET among all ages is 0.4% - 0.9% (i.e., 400 - 900 per 100,000) [22]. The prevalence of all other forms of cerebellar degeneration, combined, is approximately 20 per 100,000, which would mean that ET is 20 to 45 times more prevalent than all other forms of cerebellar degeneration combined.

### Caveats

In this article, we make the argument that ET is the most common form of cerebellar degeneration. We are not making the claim that ET is always the result of cerebellar degeneration. ET is likely a family of diseases rather than a single entity [176]. Cerebellar degeneration can result in ET but other pathophysiological mechanisms are possible and are likely. Thus, as noted above, in a subset of ET cases (8 of 33 in one reported series), the main pathology was brainstem Lewy bodies [144, 154], and in a recent report, the spectrum of observable pathology in the cerebellum in a small proportion of ET cases overlapped with that seen in controls [45]. Whether degeneration may be present in other cerebellar outflow pathways in some ET cases remains to be explored. But even with the caveat that ET is likely not always the result of cerebellar degeneration, in the majority of cases, this is what is found on postmortem studies.

### Conclusions

The degenerative cerebellar ataxias comprise a heterogeneous group of disorders whose hallmark clinical feature is ataxia, and this is accompanied, to variable degrees, by additional clinical features that are referable to cerebellar dysfunction [1–6]. ET is an exceptionally common neurological disease [22] whose primary motor feature is action tremor [23], although patients often manifest intention tremor [24–26], mild gait ataxia [27] and other features of cerebellar dysfunction [28]. In this paper, we review the abundant evidence derived from clinical, neuroimaging and postmortem studies, linking ET to cerebellar dysfunction. Furthermore, we review the combination of clinical, natural history and postmortem features that suggest that ET is neurodegenerative. We then compared the prevalence of ET to the other cerebellar degenerations and conclude that ET is 20 to 45 times more prevalent than all other forms of cerebellar degeneration combined. Given these data, it is logical to conclude that ET is, by far, the most common form of cerebellar degeneration.

Disease burden and public health impact are not solely a function of disease prevalence. ET is generally less devastating and is milder than most other forms of cerebellar degeneration, many although not all of which are associated with substantial reductions in life expectancy; nonetheless, tremor in advanced ET cases is severe and debilitating [177–179], ET is associated with a poorly-understood dementia [89], and in the limited literature, it is characterized by a modest increase in risk of mortality [175, 180]. Hence from multiple perspectives (i.e., disease prevalence, disease burden) the public health impact of ET is substantial.

## Data Availability

This is a review paper and this is not applicable.

## Abbreviations

ET: Essential tremor
MR: Magnetic resonance
PET: Positron emission tomography
SCA: Spinocerebellar ataxia
